# Case Report: A case study of positive doping control by animal-to-human drug transfer after an athlete administered medicine in spray format, containing clostebol acetate, to a pet dog

**DOI:** 10.3389/fspor.2024.1480373

**Published:** 2024-12-11

**Authors:** Andrzej Pokrywka, Dariusz Sitkowski, Olga Surała, Laurie Gheddar, Pascal Kintz

**Affiliations:** ^1^ Department of Biochemistry and Pharmacogenomics, Medical University of Warsaw, Warsaw, Poland; ^2^ Department of Physiology, Institute of Sport - National Research Institute, Warsaw, Poland; ^3^ Department of Nutrition Physiology, Institute of Sport - National Research Institute, Warsaw, Poland; ^4^ X-Pertise Consulting, Mittelhausbergen, France.

**Keywords:** adverse analytical finding, nonintentional doping, clostebol, hair analysis, contamination

## Abstract

The presence of a doping substance in an athlete's biological sample may not be only related to intentional pharmacological support. The unintended use of a prohibited substance may be due various reasons. This paper describes the case of a Polish canoeist preparing for the 2024 Summer Olympics in Paris who presented a positive doping test result, as a consequence of administering medication to her injured dog. The athlete used a Trofodermin cutaneous spray (containing clostebol acetate) for pet treatment, which resulted in human transfer during close contact and subsequent detection by doping authorities. To bolster the athlete's defense, it was essential to substantiate the scenario of an unconscious violation of anti-doping rules with scientific evidence. Hence, the decision was made to analyze and compare samples of the athlete's hair and her dog's fur. This investigation confirmed that clostebol absorption occurred through the skin of the hands, transfer during sleeping with the dog on the same bedding and/or inhalation (during the application of the medication, which was dispensed to the animal's paws). This defense was accepted by the Court of Arbitration for the Sport Anti-Doping Division, which subsequently found that the athlete committed an anti-doping rule violation, but under circumstances that amounted to a “no fault” scenario.

## Introduction

The presence of a substance or its metabolites, prohibited by the World Anti-Doping Agency (WADA), detected in the athlete's urine or blood may constitute an anti-doping rule violation (ADRV). However, the presence of a forbidden substance in an athlete's biological sample may be deliberate, due to intentional doping through pharmacological support, or unintentional. The unintended use of a prohibited substance could arise from the consumption of food products, e.g., dietary supplements contaminated or adulterated with doping agents (1), products containing hemp (cannabis) extract with tetrahydrocannabinol (THC) or poppy seeds with morphine as a natural ingredient (2). Another scenario is the consumption of contaminated meat products, where the animal was slaughted illegally or from animals treated with anabolic agents (3).

Athletes may also experience other forms of passive exposure to a substance, such as smoking e.g., crack (containing cocaine), ice or crystal meth (methamphetamine), and marijuana or hashish (containing THC), possibly resulting in positive anti-doping test results (4). However, in 2013, WADA made the decision to increase (by ten times) the threshold level allowed for carboxy-THC, and this meant that many athletes were able to avoid ADRVs, including those resulting from passive smoking. In addition, the use of drugs for medical purposes can increase the presence of prohibited compounds in the body, even after metabolization of permitted compounds to forbidden agents, e.g., codeine to morphine (5), oxethazine to phentermine and mephentermine (6), or lomerizine to trimetazidine (7).

Others have described generic pharmaceuticals as another source of diuretic contamination (8). Likewise, the use of prolonged-release drugs, drugs with a very long half-life time, or drugs which may accumulate in body tissues, could lead to inadvertent ADRVs (9). In recent years, the number of positive cases has increased due to passionate kissing and/or sexual intercourse, during which prohibited substances taken by partners enter the athlete's body (10, 11). Some have also described cross-contamination via sweat among athletes sharing the same neoprene hamstring sleeve (12). Moreover, assisting in the application of medicines, even if only by rubbing an ointment into the partner's back or administering drugs to an animal, can result in the presence of a prohibited substance in the athlete's body. For athletes, there are severe consequences for an ADVR, such as disqualification penalties and loss of income, and potentially adverse health effects or negative effects on sporting performance.

Therefore, it is crucial for an athlete to be able to prove their innocence, and, in some cases, the analysis of hair samples is a vital tool to achieve this. Although hair is not yet a routine specimen for the WADA, it is accepted in most courts of justice worldwide. Hair testing for drugs to document exposure is receiving increasing attention from scientists and lawyers, due to its long detection window, particularly compared to blood and urine, ease of collection and high compliance, and its suitability for storage at ambient temperatures. By providing information on exposure to drugs over time, hair analysis may help verify self-reported histories of drug contamination in any situation, where a history of past exposures rather than recent drug use is desired. Hair analysis can also provide a retrospective calendar of an individual's drug use. For this, multi sectional analysis is required and involves taking a length of hair and cutting it into sections to measure drug use during shorter periods of time. Given an average growth rate of 1 cm per month, each cm of hair in the vertex region represents what has circulated in the body during the corresponding month (13). In 1995, the Society of Hair Testing (SoHT) was established and published the first statement concerning the examination of drugs in human hair in 1997. Over the past 25 years, the SoHT has published consensus statements and guidelines on the best practices in hair testing, which are widely available to its members and the international community via the website (www.soht.org) (14).

In this paper, we describe the case of a Polish canoeist preparing for the 2024 Summer Olympics in Paris, who presented a positive doping test result after administering medication to her injured dog. After informing the athlete of the purpose and risks related to this publication, and allowing a sufficient reflection time of >1 week, written informed consent was obtained from the athlete for preparing this case report and any accompanying results and images.

## Case description

A month prior to the start of the 2024 Olympic Games, during a training camp in Sabaudia (Italy), the athlete was subjected to doping control. A urine sample was collected on 27th June 2024, ending at 09:26 am. On 15th July, the athlete received a notification of an Adverse Analytical Finding (AAF) and was provisionally suspended. The athlete's urine sample was found to contain clostebol metabolite (4-chloro-3a-hydroxy-androst-4-en-17-one) at an approximate concentration of 1.7 ng/ml. Notably, 20 days before the control in Sabaudia, the athlete was also subjected to a doping control during the Polish Championships, and the test result was negative. Since the athlete was in a training camp (in Italy) during the check-up, the source of the AAF was believed to be the usage of the drug Trofodermin®. Trofodermin® is a pharmaceutical preparation containing 0.5% clostebol acetate and 0.5% neomycin sulfate. It is available as a cream or spray, and is used for treating various skin conditions, such as abrasions and erosions, injuries, and wounds (15). In Italy, the general use of this drug is quite extensive.

**Figure 1:**
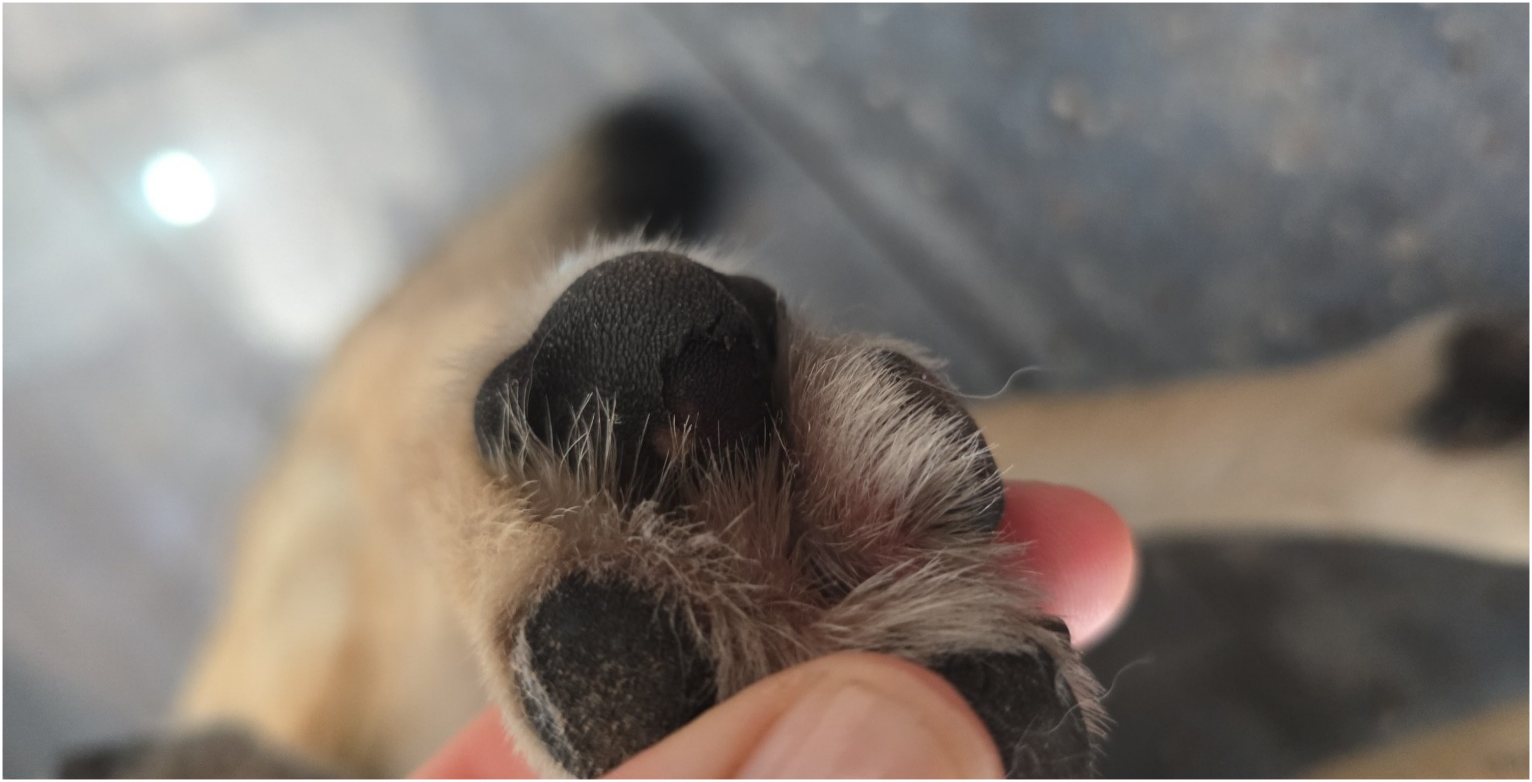
Injured at the pads of the athlete's dog.

Investigations by team management revealed that the athlete had recently used a Trofodermin® cutaneous spray (clostebol acetate, 5 mg/ml), but not for her own needs, but rather to treat wounds suffered by her pet dog (Figure 1). This medication was recommended by a local veterinarian and purchased at a local pharmacy, which the athlete had documentation to prove. The medicine was sprayed on the dog's three paws twice a day, both morning and evening, from June 19 to June 25 (inclusive). Each spray lasted approximately 2 s. The last dose was administered at approximately 10.00 p.m. on June 25.

Despite the documented evidence regarding the course of treatment for the athlete's dog, to bolster the athlete's defense, it was critical to substantiate the scenario of an unconscious violation of anti-doping rules with scientific evidence. Hence, the decision was made to analyze samples of the athlete's hair and her dog's fur. The collection of hair and fur was performed on 18th July 2024 at a laboratory in Strasbourg. Clostebol acetate was tested for identification by liquid chromatography coupled to tandem mass spectrometry (LC-MS/MS; Waters XEVO TQS micro), after methanol extraction according to a standardized laboratory procedure (16). Briefly, 30 (dog) to 100 mg (athlete) mg of finely cut hair, previously decontaminated by two dichloromethane baths (5 ml, 2 min) were weighed. An internal standard (1 ng of testosterone-d3) was added, together with 1 ml of methanol, before a 90 min ultrasonic bath at room temperature. After sample centrifugation, the organic phase was collected and evaporated. Finally, 30 µl of methanol was added and 2 µl submitted for injection onto the LC-MS/MS system.

**Figure 2:**
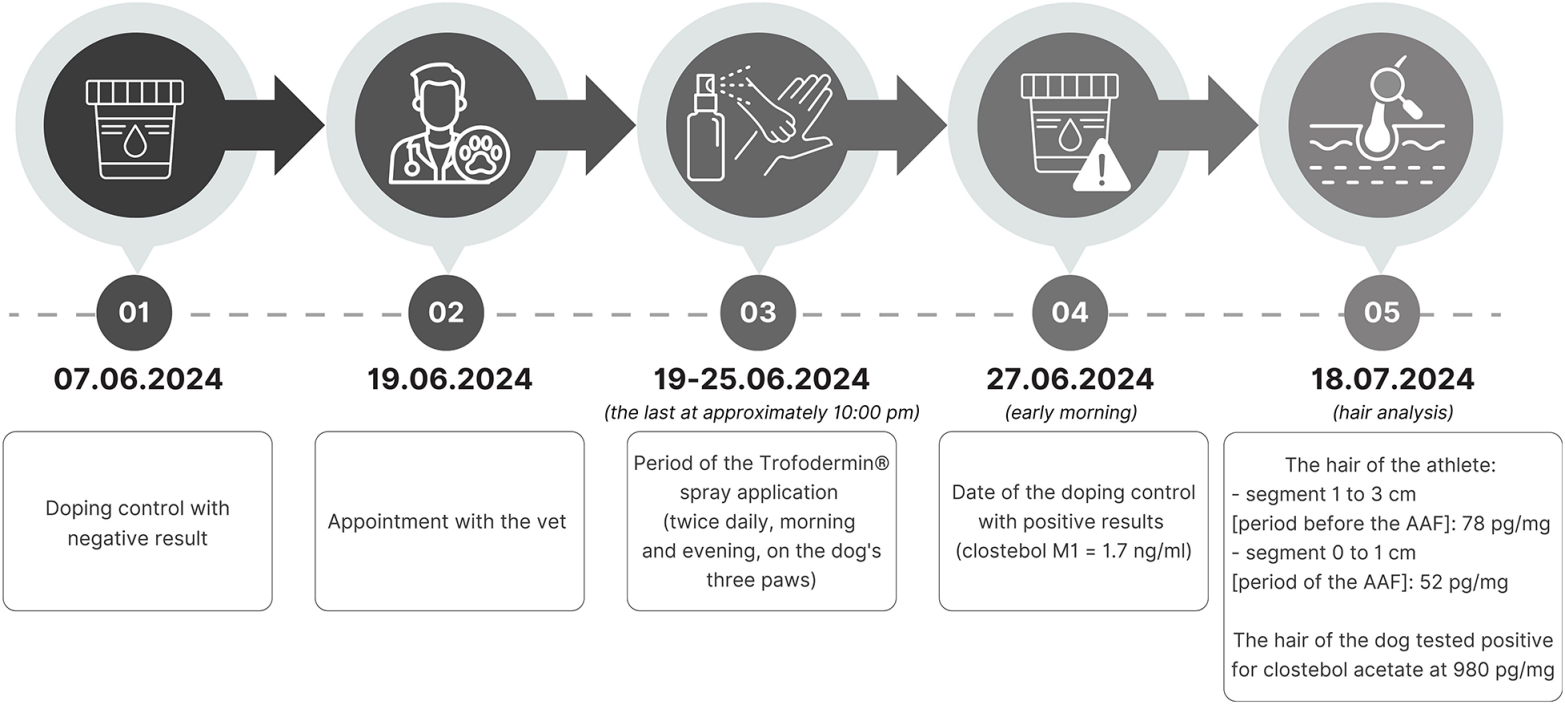
Timeline of the case.

**Figure 3:**
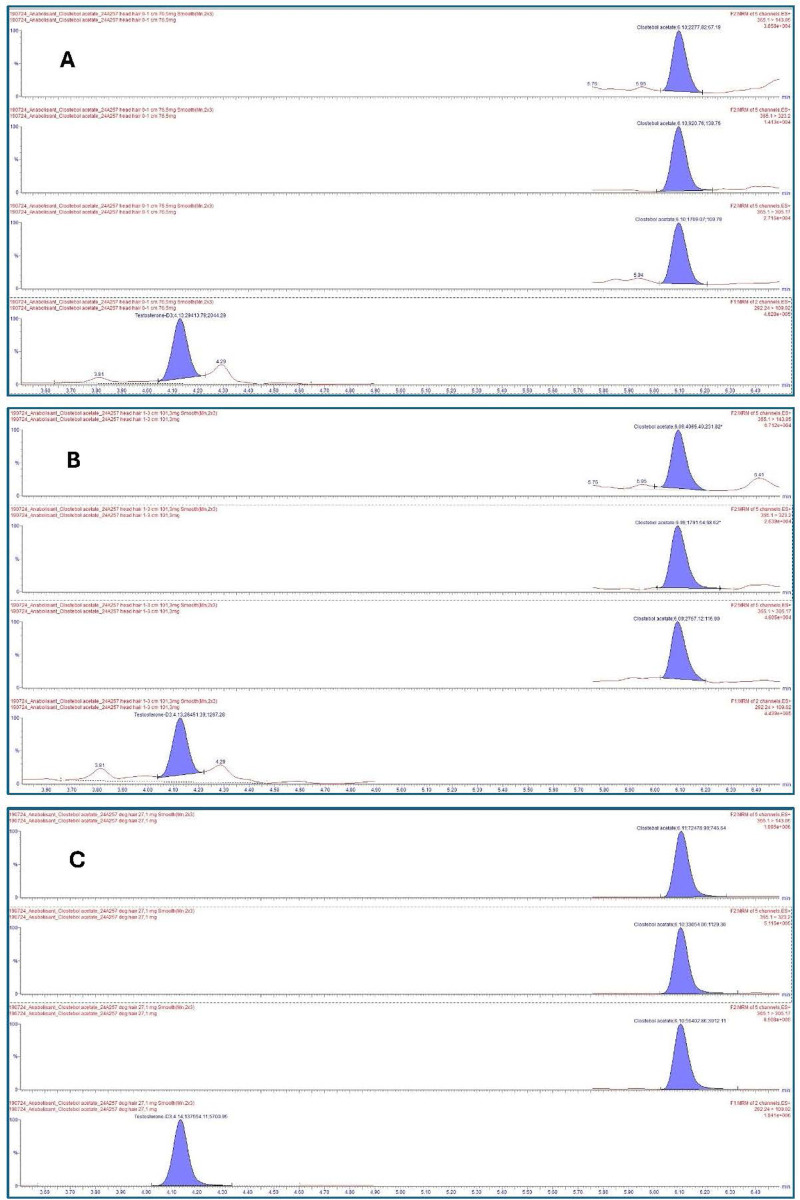
Chromatograms after extraction of **(A)** hair segment 0–1 cm of the athlete; **(B)** hair segment 1–3 cm of the athlete; and **(C)** hair of the dog. From top to bottom, 3 transitions of clostebol acetate and 1 transition of the internal standard (testosterone-D3).

In the paper published by Salomone et al. (16), it was clearly demonstrated that the ester form is the target drug after Trofodermin® exposure. For the athlete, the following results for clostebol acetate were obtained: segment 0–1 cm (period during exposure; period of the AAF when considering a hair growth rate of 1 cm per month): 52 pg/mg; segment 1–3 cm (an extended period before exposure—the 2 previous months; period before the AAF): 78 pg/mg. The dog's fur also tested positive for clostebol acetate at 980 pg/mg. The timeline of this case with relevant information is shown in Figure 2. Chromatograms of the positive clostebol acetate findings, both for the athlete and her dog, are displayed in Figure 3.

## Discussion and conclusions

Clostebol (4-chloro-testosterone; 4-chloro-4-androsten-17β-ol-3-one) is an anabolic androgenic steroid (AAS), the 4-chloro derivative of testosterone, and according to WADA rules, it is prohibited at all times and belongs to the S1.1 class (AAS). The detection of clostebol intake is traditionally based on the detection of its main metabolite (4-chloro-4-androsten-3α-ol-17- one), excreted as glucurono-conjugate in urine. However, as demonstrated 20–30 years ago, the presence of a clostebol metabolite in an athlete's urine sample can result not only from the direct use of this illegal anabolic agent, but also indirectly from the consumption of contaminated meat (17) or after drug transfer during sexual relationships with persons treated with Trofodermin® for gynecological problems (18). In recent years, improvements have been made in the detection capabilities of most anti-doping laboratories, leading to a moderate increase in clostebol detection worldwide, and especially in Italy, as already mentioned.

Before the Rio de Janeiro 2016 Summer Olympic Games, several Olympic athletes tested positive for clostebol, where the main metabolite of clostebol (4-chloro-4-androsten-3α-ol-17-one) was detected at relatively low concentrations (ca. 1 ng/ml). In some instances, the athletes declared not using the cream during a hearing process, but recognized possible exposure after applying this cream to a teammate. Scientific literature now provides strong corroborating evidence that a positive anti-doping test result, due to accidental contact with other clostebol users or via other sources, is indeed very likely.

The transdermal application of clostebol acetate can produce detectable amounts of metabolites in urine, even after a single exposure. Depending on the protocols, the main clostebol metabolite (4-chloro-androst-4-en-3α-ol-17-one, M1) was found to be detectable up to 30–40 ng/ml (at peak concentration) for more than 10 days. In other studies, the transfer of clostebol from one subject to another occurred during hand shaking or sexual intercourse (15, 18–20). In those cases of drug transfer between two humans, as expected, urine concentrations of the clostebol metabolite M1 were lower vs. those cases of direct ingestion, but generally not exceeding 1–5 ng/ml.

In order to generate guidelines that could inform sporting authorities when reviewing such cases, De la Torre et al. (15) reviewed evidence of Phase I and Phase II clostebol metabolism. The main clostebol metabolite M1 generally used at the screening level, as well as three other metabolites (M2–M4), were mainly excreted as glucuronides, whereas M5 (4ζ- chloro-5ζ-androstan-3β-ol-17-one) is predominantly excreted as sulfate. Unfortunately, neither 5α-reductase activity (impaired by the presence of the chlorine in C4) nor specific sulfotransferases present in the skin, allowed for a clear distinction of the administration route. According to the authors, studies with a larger number of volunteers, and investigating other physiological fluids allowed in antidoping (e.g., blood), are needed. The information gained could help establish a reporting level for M1, maybe creating some false negatives, but excluding nonintentional doping scenarios. In their final conclusions, the authors stated that the detection of clostebol's main metabolites is not an unusual finding in sports drug testing in Italy, when compared with other areas worldwide. The main reason is that pharmaceutical formulations containing clostebol are common in this country and can be obtained over the counter without a medical prescription; hence, the risk of their unintentional ingestion is much greater in Italy than elsewhere.

As mentioned before, the athlete used a drug recommended by a veterinarian to treat her dog, and this was purchased from a local pharmacy. This case is unprecedented, because it involves the use of Trofodermin not in cream form (the source of many unintentional anti-doping rule violations), but in spray form. Therefore, clostebol could have been absorbed both through the skin of the hands, while rubbing the medication into the dog's paws, and via product inhalation. The athlete was in close contact with her dog during the whole camp, including the period between 19 and 27 June (she stayed in the same room with him during the camp). The athlete had direct contact with the dog every day, including episodes of hugging, touching, wound checking, the dog licking the athlete's face/cheeks, and lying in the same bed, where the dog slept with the athlete every night. The living conditions of the athlete are another relevant factor. She stayed in a hotel room with air conditioning on, due to the hot weather in Italy at that time, and rarely opened the windows (preventing natural air circulation), which meant a confined living space with her dog. It is worth noting that a similar case of unintentional doping rule violation, via the application of medication to an animal, was reported in 2022 and involved cyclist Katerina Nash. The U.S. Anti-Doping Agency also found that the athlete showed no fault or negligence (21).

Due to the use of a spray rather than an ointment or cream, the direct reference of this case to any already described in the scientific literature was impossible. Thus, the athlete took advantage of the possibility of having her hair samples, as well as the dog's hair, tested at a centre run by Professor Pascal Kintz, whose research, including that on clostebol, has helped in many cases to substantiate or prove unintentional anti-doping rules’ violations (16, 22). By also providing information on drug contamination over time, hair analysis may help verify self-reported histories of drug use in any situation, and it can provide a retrospective calendar of an individual's drug exposure history. For this purpose, multi-sectional analysis is required, which involves taking the length of hair and cutting it into sections to measure a target metabolite during shorter periods. Given an average growth rate of 1 cm per month, each cm of hair in the vertex region represents what has circulated in the body during the corresponding month (13). In the case described, such an analyses could answer questions around whether the athlete ingested a therapeutic dose of clostebol, either intentionally or unintentionally, and if the positive doping test was due to contamination related to some medicinal purpose (e.g., treatment of an animal).

The finding of similar clostebol concentrations, between the two segments of the athlete's hair, are more consistent with contamination from spray contact and/or the environment (such as sleeping with the dog on the same bedding and pillow). These concentrations demonstrate that the athlete was in close contact with the dog, even for a short period. The dog was sprayed with medicine and then slept with the athlete. This resulted in an initial transfer of clostebol to the pillow and bedding, followed by a second transfer from the pillow and bedding to the athlete. A similar situation has been described for child, regarding propranolol and quetiapine contamination, where the donor was a long-term repetitive user of both substances (23). The concept of environmental contamination and doping has also been published in relation to cocaine (24). Finally, it is reasonable to consider that after spraying Tofodermin® on the dog, some drug remained on the athlete's hand, and the athlete touched her hair multiple times a day, which could result in several segments testing positive, as they did.

With this limited study, it is unclear as to whether the animal-to-human transfer process outlined can be applied to other nonintentional doping scenarios. Future research should examine clostebol excretion in the urine of those people using different drug formations and their administration to other animals. As mentioned in the introduction, hair is not yet a routine specimen for the WADA, but is accepted in most courts of justice in the world. The SoHT was established in 1999 during its annual meeting, a consensus on hair testing for doping agents. The statement includes that hair specimens are not suitable for general routine control and that a negative hair result cannot exclude the administration of the detected drug and cannot overrule the AAF (25). However, a negative hair test result is also a result. This can be interpreted in two different ways: 1 the owner of the hair did not take, or was not exposed to, the specific drug, or 2 the procedure is not sensitive enough to detect the drug. Further difficulties with interpretation can arise from a suspected single drug exposure, whereas repeated exposures over time will likely favor identification by hair analysis. Another limitation is the minimal detectable dosage (of a target compound) in hair. The analytical method of choice needs to be sensitive enough to identify traces of drugs, such as cases where an athlete's urine specimen returns a positive result and the hair sample returns a negative result (26).

Another challenge is that hair samples can be manipulated and/or degraded through cosmetic or hair treatments, potentially altering drug concentrations which could eventually lead to a false negative test result. In particular, the oxidative bleaching of hair samples under alkaline conditions has a significant effect on some drug concentrations. The alteration of hair by cosmetics can be documented (27), and in the present study, evidence was provided (and verified) to support the athlete's case of non-intended use of a prohibited compound.

Leaving aside these limitations, the authors of this manuscript express the belief that the case described can influence anti-doping policy. To avoid tedious discussion about possible contamination, it is not unreasonable to establish a reporting level for clostebol metabolite M1. The authors suggest that WADA evaluate the possibility of urine test results as atypical findings, as is the case for clenbuterol, ractopamine, zilpaterol, or zeranol (28), where the concentration does not exceed 1 or 2 ng/ml. This corresponds to data published by de la Torre et al. (15). Based on internal data of the anti-doping laboratory in Rome, for the period 2003–2018, 47 cases were reported (40 of them after 2013) with concentrations below 2 ng/ml in 77% of the cases. These observations and the athletes’ reports during the hearings, claimed that the use of Trofodermin® cream or contact with teammates using the cream was the main source of clostebol metabolites present in urine, after the transdermal application of clostebol acetate in different individuals (15).

The authors from France have considerable experience with hair analysis, which has often shed light on the origin and frequency of licit and illicit substances. In the case of clostebol exposure from skin contamination, the hair concentration is expected to be in the low pg/mg range, or even undetectable (16). Instead, the hair collected from individuals who contaminated the athlete, after having used Trofodermin© themselves, will likely result in a higher concentration, as reported in this case.

In conclusion, the athlete and her legal team mounted a strong defense that turned a positive urine result into a case of unintended use, based on contact with the Trofodermin® spray to treat her dog. Supporting evidence included; 1 a very low concentration of clostebol metabolite (1.7 ng/ml) in the urine sample of the athlete with AAF, 2 the negative result of the doping control conducted 20 days earlier, 3 as well as a significantly higher level of clostebol in the animal's fur (980 pg/mg) than in athlete's hair (52 pg/mg), 4 along with the facts presented by the athlete, including the treatment of her dog with Trofodermin® spray. The absorption of clostebol, a component of this medication, occurred through the skin of the hands, drug transfer during sleeping with the dog on the same bedding and/or inhalation. Ultimately, the Court of Arbitration for Sports Anti-Doping Division (CAS ADD) accepted this defense, which amounted to a “no fault” scenario. Two days later, the athlete started her first race during the 2024 Summer Olympics Games in Paris.

## Data Availability

The data analyzed in this study is subject to the following licenses/restrictions: data includes sensitive testing results (from routine doping control) which cannot be publicly shared. Requests to access these datasets should be directed to andrzej.pokrywka@wum.edu.pl.
